# Maintenance of somatic tissue regeneration with age in short‐ and long‐lived species of sea urchins

**DOI:** 10.1111/acel.12487

**Published:** 2016-04-20

**Authors:** Andrea G. Bodnar, James A. Coffman

**Affiliations:** ^1^ Bermuda Institute of Ocean Sciences17 Biological StationSt. George'sGE01Bermuda; ^2^ MDI Biological Laboratory159 Old Bar Harbor RoadSalisbury CoveME04672USA

**Keywords:** negligible senescence, regeneration, sea urchin, Seawi, tissue homeostasis, Vasa

## Abstract

Aging in many animals is characterized by a failure to maintain tissue homeostasis and the loss of regenerative capacity. In this study, the ability to maintain tissue homeostasis and regenerative potential was investigated in sea urchins, a novel model to study longevity and negligible senescence. Sea urchins grow indeterminately, regenerate damaged appendages and reproduce throughout their lifespan and yet different species are reported to have very different life expectancies (ranging from 4 to more than 100 years). Quantitative analyses of cell proliferation and apoptosis indicated a low level of cell turnover in tissues of young and old sea urchins of species with different lifespans (*Lytechinus variegatus*,* Strongylocentrotus purpuratus* and *Mesocentrotus franciscanus*). The ability to regenerate damaged tissue was maintained with age as assessed by the regrowth of amputated spines and tube feet (motor and sensory appendages). Expression of genes involved in cell proliferation (*pcna*), telomere maintenance (*tert*) and multipotency (*seawi* and *vasa*) was maintained with age in somatic tissues. Immunolocalization of the Vasa protein to areas of the tube feet, spines, radial nerve, esophagus and a sub‐population of circulating coelomocytes suggests the presence of multipotent cells that may play a role in normal tissue homeostasis and the regenerative potential of external appendages. The results indicate that regenerative potential was maintained with age regardless of lifespan, contrary to the expectation that shorter lived species would invest less in maintenance and repair.

## Introduction

Aging is characterized by a failure to maintain tissue homeostasis, resulting in a decline in tissue function and a diminished response to damage (Rando, 2006). Age‐related tissue decline is not limited to mammals but is also experienced by some highly regenerative vertebrates (Tsai *et al*., 2007; Anchelin *et al*., 2011; McCusker & Gardiner, 2011; Kaslin *et al*., 2013; Seifert & Voss, 2013). Somatic or tissue‐specific stem cells maintain tissues in adult organisms of many species and can replace cells during normal homeostasis or in response to injury or disease. Intrinsic changes within stem cells, alterations within the local stem cell niche, and/or changes in the surrounding tissues or systemic factors that affect stem cell function are thought to underlie the age‐related decreased capacity for repair and regeneration (Rando, 2006).

Sea urchins are highly regenerative animals with indeterminate growth and life‐long reproduction, and thus offer the opportunity to investigate mechanisms for long‐term maintenance of tissue homeostasis and regenerative capacity. In addition, different species of sea urchins have very different reported life expectancies, offering an opportunity to investigate the role of regenerative processes in lifespan determination. The red sea urchin [*Mesocentrotus franciscanus*; formerly known as *Strongylocentrotus franciscanus* (Kober & Bernardi, 2013)] is one of the earth's longest living animals, living well in excess of 100 years with no age‐related increase in mortality rate or decline in reproductive capacity (Ebert & Southon, 2003; Ebert, 2008). In contrast, the maximum life expectancy of *Lytechinus variegatus* in the wild is estimated to be only 4 years (Moore *et al*., 1963; Beddingfield & McClintock, 2000) while that of *Strongylocentrotus purpuratus* is estimated to be more than 50 years (Ebert, 2007, 2010). Although *S. purpuratus* and *M. franciscanus* are native to the Pacific Ocean and *L. variegatus* is found in the Atlantic, they have similar lifestyles as herbivorous grazers in near‐shore environments. Key differences that may have a role lifespan determination relate to their different reproductive strategies, with the longer lived species having a later onset of sexual maturation and a longer planktonic larvae phase with low and variable recruitment into adults (Ebert *et al*., 1994). In addition, the longer lived species have thicker tests (shells) and spines and an expansion of genes associated with innate immunity (Buckley & Rast, 2012) indicating a greater investment in these key defense mechanisms.

Sea urchins are known to regenerate external appendages such as spines and tube feet (Candia Carnevali, 2006). Spines are tapered skeletal elements made of magnesium‐containing calcite embedded in the dermis and covered by an epidermis (Dubois & Ameye, 2001; Politi *et al*., 2004). Spine regeneration involves a wound‐healing process where the epidermis is reconstituted around the broken spine and biomineralization is directed by the skeletogenic cells (sclerocytes) located in the dermis (Dubois & Ameye, 2001; Politi *et al*., 2004). Tube feet are fleshy external appendages that protrude through the sea urchin shell and play a role in locomotion, respiration, and sensory perception (Lesser *et al*., 2011). There are about 1500 tube feet per sea urchin and each is a cylindrical structure composed of multiple tissue layers: an outer epidermis, a basiepidermal nerve plexus, a connective tissue layer, and a longitudinal muscle layer lined with ciliated epithelium facing the inner water vascular lumen (Lesser *et al*., 2011; Santos *et al*., 2013). A disc at the distal end of each tube foot is used for adhesion and also receives sensory input which is transduced to the radial nerve that lies just inside the shell (Lesser *et al*., 2011). Tube feet regeneration has not been investigated in detail, but has recently been shown to require Notch signaling (Reinardy *et al*., 2015). In other echinoderms, tissue regeneration appears to employ both epimorphic and morphallatic processes (Candia Carnevali, 2006). Studies of arm regeneration in the sea star *Asterias rubens* suggest that the proliferating cells originate from undifferentiated progenitor cells (Hernroth *et al*., 2010); however it is not known whether true adult stem cells and distinct stem cell niches exist in somatic tissues of echinoderms.

The objectives of this study were to measure tissue homeostasis and regenerative potential in sea urchin tissues, to determine if these processes are different in species with different lifespans, and if they are affected by age. This was achieved by characterizing cell turnover and tissue regeneration in sea urchins with respect to age and measuring markers of cell proliferation and stem cell function. For this, the expression of *pcna*,* tert*,* vasa*, and *seawi* was investigated at the mRNA level, and Vasa protein was investigated by immunofluorescence. PCNA (Proliferating Cell Nuclear Antigen) is an essential component of the DNA replication machinery and serves as a marker for cell proliferation (Moldovan *et al*., 2007). TERT (Telomerase Reverse Transcriptase) is the catalytic component of telomerase, an enzyme that maintains telomeres and genomic integrity and thus plays an important role in cell proliferation and tissue homeostasis (Daniel *et al*., 2012). Vasa is a DEAD‐box RNA helicase that acts as a translation regulator, but it has also been shown to play a role in pre‐mRNA splicing, ribosome biogenesis and nuclear export (Gustafson & Wessel, 2010). Seawi is the sea urchin Piwi homolog, belonging to the highly conserved Piwi/Argonaute family that binds to specific micro‐RNAs called piRNAs which act in transposon silencing and regulation of transcriptional activity (Palakodeti *et al*., 2008). Vasa and Piwi play a role in germline development and maintenance in *Drosophila melanogaster*,* Caenorhabditis elegans*, and mammals (mice and humans). However, in other animals (e.g., cnidarians, planarians, tunicates), their expression is not restricted to the germline but is also found in multipotent stem cells that are capable of producing both somatic and germline derivatives (Gustafson & Wessel, 2010; Juliano *et al*., 2010). As many of the animals that express Piwi and/or Vasa in their multipotent cells possess a high regenerative capacity in their tissues, the presence of these proteins in the tissues of adult sea urchins would support the existence of multipotent cells that provide somatic regenerative capacity.

## Results

### Age estimates for sea urchins

Sea urchins of each species were divided into two age groups; young and old. The average test (shell) diameter, the number of animals in each group and estimated ages are shown in Table 1. Ages of individual sea urchins were estimated from test diameter using previously established growth curves (see Experimental procedures). As growth rate can be affected by environmental factors (e.g., food availability), the ages are only estimates. However, animals at the upper and lower size range of their respective populations were selected for this study, and the relative size of animals collected at the same time and place is reasonably indicative of relative age within the population.

**Table 1:** Estimated ages of young and old *Lytechinus variegatus*,* Strongylocentrotus purpuratus*, and *Mesocentrotus franciscanus* used for cell proliferation, apoptosis, gene expression, immunostaining, and tissue regeneration

Sample	Test diameter (mm)	Est. age (years)	Reference
Cell proliferation (BrdU incorporation) and apoptosis (Apo ssDNA)			
Lv – Young Lv – Old	33.8 ± 1.0 (6) 70.2 ± 2.1 (6)	1.1 ± 0.1 (6) 3.8 ± 0.2 (6)	Beddingfield & McClintock (2000)
Sp – Young Sp – Old	33.5 ± 0.6 (6) 76.6 ± 1.1 (6)	2.0 ± 0.1 (6) 53.3 ± 6.9 (6)	Coleman (1993)
Mf^a^ – Young Mf^a^ – Old	36.2 ± 0.7 (6) 115.8 ± 4.3 (6)	3.2 ± 0.1 (6) 20.1 ± 3.8 (6)	Ebert (1998) Ebert *et al*. (1999)
Gene expression, immunohistochemistry and immunocytochemistry and TUNEL			
Lv –Young Lv – Old	31.8 ± 2.5 (6) 62.8 ± 0.8 (6)	1.1 ± 0.1 (6) 3.2 ± 0.1 (6)	Beddingfield & McClintock (2000)
Sp – Young Sp – Old	36.9 ± 1.0 (6) 75.9 ± 1.0 (6)	2.2 ± 0.1 (6) 49.3 ± 6.0 (6)	Coleman (1993)
Mf^b^ – Young Mf^b^ – Old	37.9 ± 2.9 (6) 99.3 ± 0.8 (6)	3.3 ± 0.1 (6) 10.6 ± 0.2 (6)	Ebert (1998) Ebert *et al*. (1999)
Mf^c^ – Young Mf^c^ – Old	42.2 ± 1.7 (6) 157.0 ± 1.5 (4)	3.5 ± 0.1 (6) 113.8 ± 7.8 (4)	Ebert (1998) Ebert *et al*. (1999)
Tube feet and spine regeneration			
Lv –Young Lv – Old	32.7 ± 1.0 (6) 68.8 ± 1.5 (6)	1.1 ± 0.1 (6) 3.7 ± 0.1 (6)	Beddingfield & McClintock (2000)
Sp – Young Sp – Old	37.6 ± 2.0 (4) 68.1 ± 2.1 (4)	2.2 ± 0.2 (4) 22.2 ± 5.4 (4)	Coleman (1993)

The values are expressed as mean ± standard error and the numbers in brackets represent the number of samples (*n*) in each group.

a *Mesocentrotus franciscanus* collected in April 2013 from California used for BrdU and Apo ssDNA.

b *Mesocentrotus franciscanus* collected in April 2013 from California, used for TUNEL and immunohistochemistry.

c *Mesocentrotus franciscanus* collected in August 2013 from British Columbia, used for gene expression and coelomocyte immunocytochemistry.

### Cell proliferation and apoptosis in sea urchin tissues

**Figure 1:**
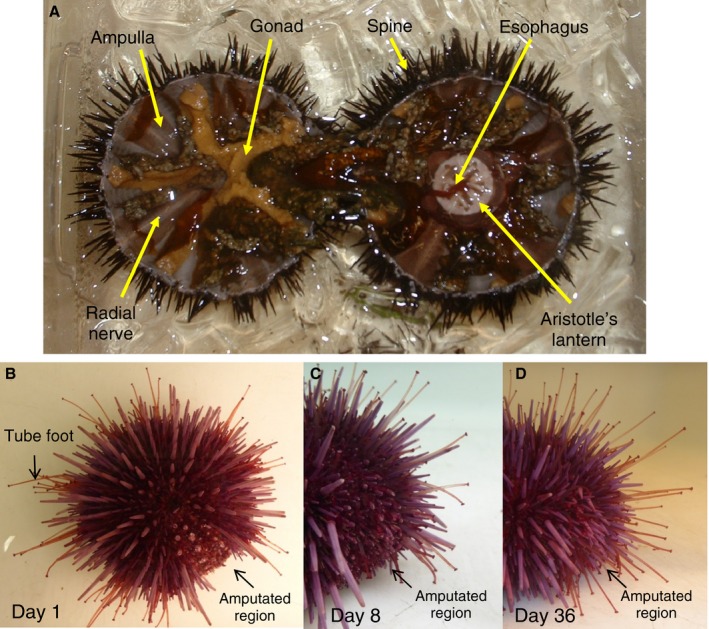
Sea urchin anatomy and tissue regeneration. (A) Cross sectional view of *Lytechinus variegatus* showing tissues used in this study: spine, gonad, muscle from Aristotle's lantern jaw structure, esophagus, radial nerve along the inside wall of the test (shell) surrounded by the ampulla, part of the water vascular system which is continuous with the tube feet that protrude through the test. Coelomocytes freely circulate in the coelomic fluid that fills the body cavity. B through D show the spine and tube feet regeneration assay using *Strongylocentrotus purpuratus*. (B) Aboral view 1 day postamputation showing tube feet and spines removed from along one of the ambulacral segments of the test from the oral to aboral surface. (C) Lateral view of the amputated region, 8 days postamputation. (D) Lateral view of the amputated region from the same animal, 36 days postamputation.

**Figure 2:**
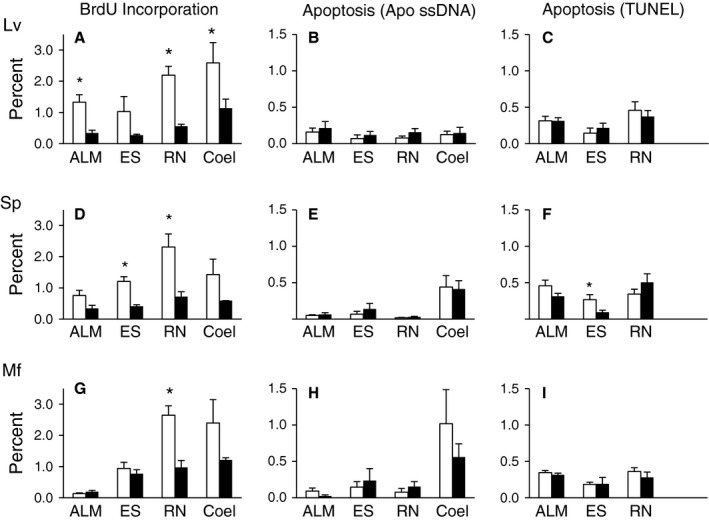
Cell proliferation and apoptosis in sea urchin tissues. Percent of cells undergoing cell proliferation (BrdU incorporation) and apoptosis (Apo ssDNA and TUNEL) in tissues of *Lytechinus variegatus* (A–C), *Strongylocentrotus purpuratus* (D–F), and *Mesocentrotus franciscanus* (G–I). The white bars represent small/young animals and the black bars represent large/old animals and the tissues investigated are: Aristotle's lantern muscle (ALM), esophagus (ES), radial nerve (RN), and coelomocytes (Coel). Age estimates and number of animals in each group are shown in Table 1. The data are presented as means and standard errors, and stars (*) indicate a significant difference between age groups (*P* < 0.05).

Cell proliferation was quantified by BrdU incorporation in tissues of three species of sea urchin with different lifespans. The tissues used for this analysis were Aristotle's lantern muscle, radial nerve, esophagus, and coelomocytes (Fig. 1A). The sizes and estimated ages of the sea urchins used for these experiments are shown in Table 1. In these collections, *L. variegatus* and *S. purpuratus* had a good size separation range between the different age groups, while the largest *M. franciscanus* were not particularly old due to difficulties finding large animals at the site of collection. Overall levels of BrdU incorporation during a 24‐h treatment period were low ranging from 0.14% to 2.65%, with the highest levels of cell proliferation in the coelomocytes and radial nerve (Fig. 2). One‐way ANOVA or Kruskal−Wallis tests and *post hoc* analyses revealed few interspecies differences in cell proliferation when compared within age categories. There was a trend for decreasing BrdU incorporation with age/size with results reaching statistical significance in muscle, nerve, and coelomocytes for *L. variegatus*, esophagus and nerve of *S. purpuratus*, and nerve of *M. franciscanus* (*P* < 0.05 for all) (Fig. 2). The total number of cells analyzed, as well as representative images are shown in Fig. S1 (Supporting information).

Apoptosis was measured using the Apo ssDNA assay, which allowed detection of apoptotic cells in the same tissue samples from BrdU‐treated animals that were used for the cell proliferation assay. Levels of apoptosis were low, with no significant differences observed between tissues from young and old animals. In *M. franciscanus* coelomocytes, the level of apoptosis ranged from 0.57% to 1.16% while in the other tissues from all species it ranged from 0.02% to 0.19% (Fig. 1). As expected for animals with indeterminate growth, the amount of cell proliferation indicated by BrdU incorporation was significantly higher than the level of apoptosis in the corresponding tissue and age group for each species in most cases (*P* < 0.05). One‐way ANOVA or Kruskal−Wallis tests and *post hoc* analyses revealed only a few interspecific differences for apoptosis when compared within tissues type and age group.

To confirm the low overall levels of apoptosis in sea urchin tissues, TUNEL was performed on tissue sections from sea urchins collected at the same time and place, but not BrdU‐labeled (Fig. 2). There were insufficient amounts of coelomocytes collected from these animals to conduct the TUNEL analysis on these cells. One‐way ANOVA or Kruskal−Wallis tests revealed no species‐specific differences in TUNEL when tissues were compared within age categories. In most cases, the level of apoptosis did not change with age. The overall levels of apoptosis measured by TUNEL ranged from 0.10% to 0.51% and, in some cases, when compared within tissue type and age category, were significantly higher than those estimated by Apo ssDNA. This is consistent with reports that the Apo ssDNA technique is more specific, with fewer false positives than TUNEL (Frankfurt & Krishan, 2001). The total number of cells analyzed, as well as representative images are shown in Figs. S2 and S3.

**Figure 3:**
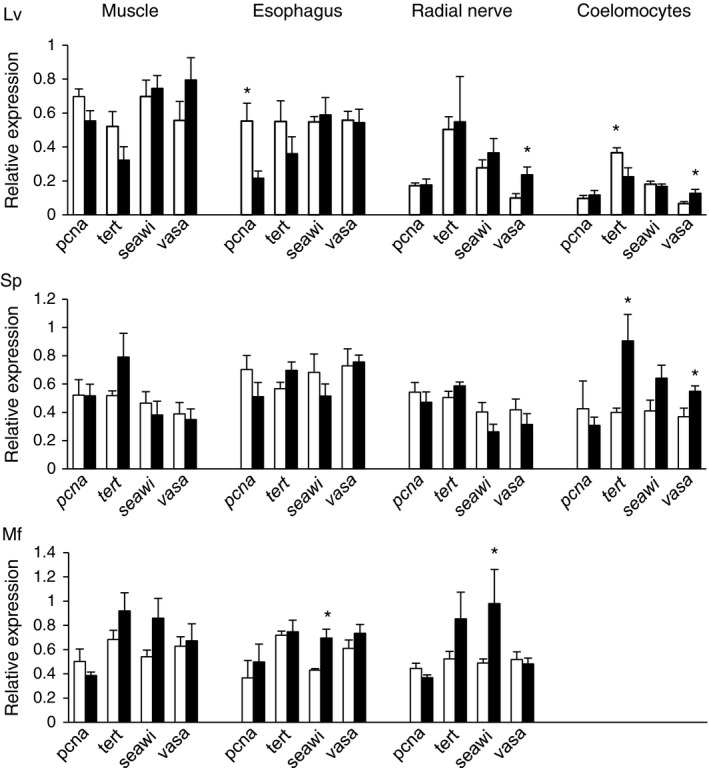
Relative gene expression for *pcna*,* tert*,* seawi*, and *vasa* in tissues of *Lytechinus variegatus* (Lv), *Strongylocentrotus purpuratus* (Sp), and *Mesocentrotus franciscanus* (Mf). The white bars represent small/young animals and the black bars represent large/old animals and the tissues investigated are: Aristotle's lantern muscle (Muscle), Esophagus, Radial Nerve, and Coelomocytes. Age estimates and number of animals in each group are shown in Table 1. Data are presented as means and standard errors, and stars (*) indicate a significant difference between age groups (*P* < 0.05).

Expression of genes involved in cell proliferation (*pcna*) and telomere maintenance (*tert*) was investigated in tissues (muscle, esophagus, radial nerve and coelomocytes) of young and old animals of each species. For these assays, *L. variegatus* and *S. purpuratus* were collected at the same time and place as the animals used for BrdU incorporation, whereas for *M. franciscanus* the collection was conducted at a different location that had a better size range of animals including much larger/older animals (Table 1). Coelomocytes from *M. franciscanus* were collected, but thawed during transport and the RNA from these tissues was of too poor quality to conduct qRT‐PCR. Overall, the expression of these genes was maintained with age with few exceptions (Fig. 3). Gene expression levels are reported as qRT‐PCR threshold cycle (Ct) values in Table S1 (Supporting information).

### Tube feet and spine regeneration in *L. variegatus* and *S. purpuratus*

**Figure 4:**
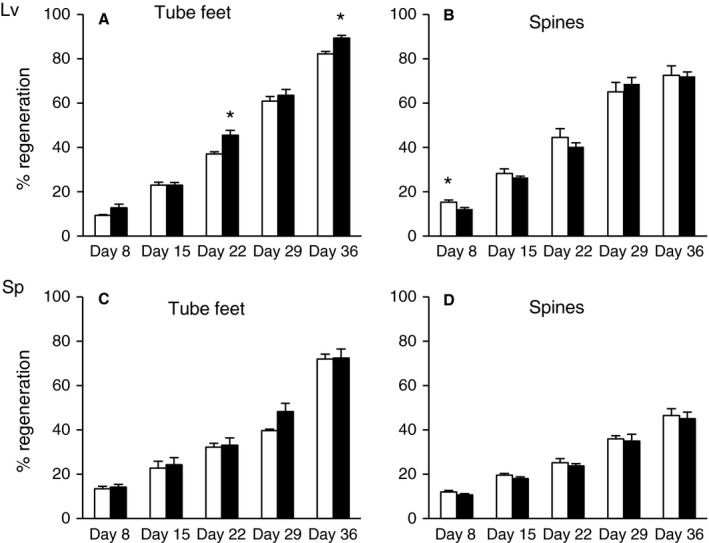
Regeneration of tube feet and spines with respect to age in *Lytechinus variegatus* (A and B) and *Strongylocentrotus purpuratus* (C and D). Regrowth of tube feet and spines was measured at 8, 15, 22, 29, and 36 days postamputation and expressed as a percentage of full‐length, nonamputated appendages. The white bars represent small/young animals and the black bars represent large/old animals. Age estimates and number of animals in each group are shown in Table 1. The data are presented as means and standard errors, and stars (*) indicate a significant difference between age groups (*P* < 0.05).

To determine whether regenerative potential was maintained with age, the regrowth of amputated spines and tube feet was measured in *L. variegatus* and *S. purpuratus* (Fig. 1B–D). The average test diameters and the estimated ages of the sea urchins used for the regeneration experiments are shown in Table 1. Regrowth of spines and tube feet was expressed as a percentage of full‐length unamputated spines and tube feet for each animal and was measured every 7 days postamputation for a 5 week period. There was a significant difference in regrowth between each time point for tube feet and spines of both species (GLM, *P* = 0), and the results demonstrated that the rate of regrowth of both spines and tube feet was maintained with age in *L. variegatus* and *S. purpuratus* (Fig. 4). A few time points for *L. variegatus* demonstrated statistically significant age effects, however the differences were small. When the experiment was repeated with an additional group of *L. variegatus*, only the slight decrease in spine regrowth with age at Day 8 postamputation was observed (data not shown).

**Figure 5:**
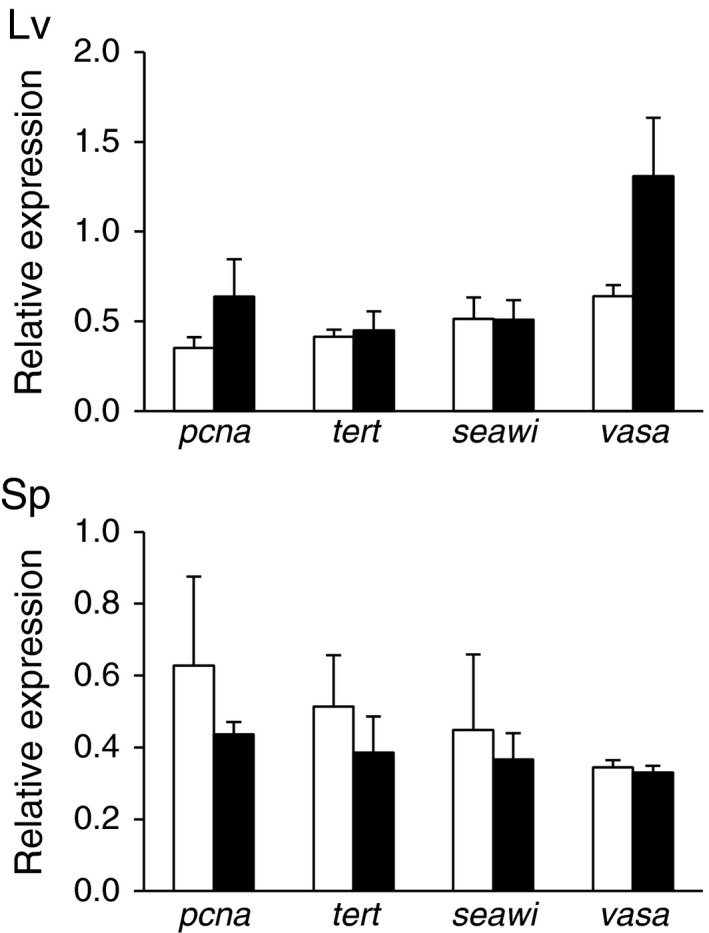
Relative gene expression for *pcna*,* tert*,* seawi,* and *vasa* in tube feet from *Lytechinus variegatus* (Lv) and *Strongylocentrotus purpuratus* (Sp). The white bars represent small/young animals and the black bars represent large/old animals. Age estimates and number of animals in each group are shown in Table 1. Data are presented as means and standard errors.

Quantitative RT‐PCR analysis indicated no age‐related changes in expression of genes involved in cell proliferation (*pcna*) and telomere maintenance (*tert*) in the tube feet amputated from these animals at the beginning of the experiment (Fig. 5, Table S1).

### Expression of multipotency genes in sea urchin tissues

The expression of three genes that are associated with germline determination and multipotency (*seawi*,* vasa,* and *nanos2*) was investigated with respect to age in sea urchin tissues. *nanos2* was highly expressed in adult gonad tissue but expressed at low levels in somatic tissues and was therefore not pursued further (qRT‐PCR Ct values > 30, Table S2). In contrast, the expression of *seawi* and *vasa* was easily measured in all somatic tissues tested, with qRT‐PCR Ct values < 30 (Table S1). Expression of these genes was maintained with age in most tissues (Fig. 3 and Fig. 5), except *vasa* expression showed a significant increase with age in radial nerve and coelomocytes of *L. variegatus* (*P* = 0.03 and *P* = 0.05, respectively) and coelomocytes of *S. purpuratus* (*P* = 0.04) (Fig. 3). The expression of *seawi* significantly increased with age in esophagus and radial nerve of *M. franciscanus* (*P* = 0.002 and *P* = 0.04, respectively) (Fig. 3). Spines and tube feet from young and old *M. franciscanus* and spines from young and old *L. variegatus* were not collected and therefore age comparisons were not possible for these tissues. However, expression of *vasa* and *seawi* was detected in RNA from spines at comparable levels to other somatic tissues for both *L. variegatus* and *S. purpuratus* (Table S1).

### Localization of Vasa in sea urchin cells and tissues

**Figure 6:**
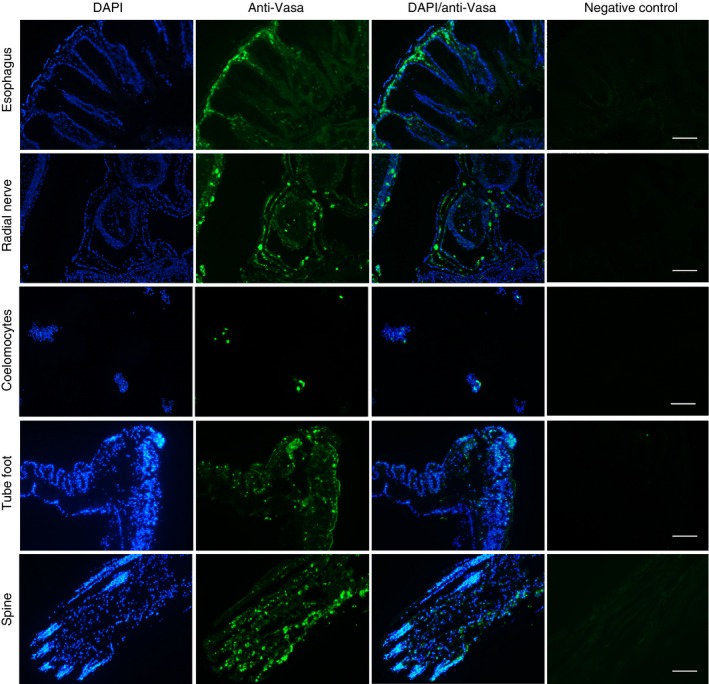
Immunohistochemistry and immunocytochemistry of sea urchin tissues and cells using an antibody to Vasa. Esophagus, radial nerve, coelomocytes, tube foot, and spine from *Lytechinus variegatus* stained with DAPI or reacted with anti‐Vasa antibody visualized with DyLight^™^ 488 secondary antibody. Negative control panels are tissue sections treated with only the secondary antibody. Scale bar is 100 μm.

Immunohistochemistry was used to localize the Vasa protein in different tissues. For these experiments, four samples of each tissue (muscle, esophagus, and radial nerve) for each species were analyzed (Table 1). Representative data for *L. variegatus* are shown in Fig. 6 and those for *S. purpuratus* and *M. franciscanus* are shown in Fig. S4. Esophagus tissue from all three species consistently showed areas of Vasa staining at the base or within the villi (Figs. 6 and S4). Radial nerve also showed areas of Vasa staining; however the specific vasa‐positive cell types could not be identified (Figs. 6 and S4). Muscle had only a low level of diffuse staining without localized areas of high signal (data shown only for *M. franciscanus*, Fig. S4). To quantify the level of Vasa staining in esophagus and radial nerve, the percent of Vasa‐positive cells was estimated from the number of nuclei surrounded by Vasa staining when images from both fluorescent channels were overlaid. This ranged from 0.9% to 4.3% for radial nerve, with lower levels of immunoreactivity in *M. franciscanus* tissue compared to the other two species. Vasa‐positive cells ranged from 2.5% to 5.1% for esophagus with no significant difference between species.

Immunocytochemistry was used to localize Vasa in the coelomocytes from all three sea urchin species. The coelomocytes used for each species were from the same young and old animals that were used for gene expression studies (Table 1). Coelomocytes from all three species showed a distinct subpopulation of Vasa‐positive cells (Figs. 6 and S4). The percent of Vasa‐positive coelomocytes ranged from 0.9% to 2.8% and did not change with age for *S. purpuratus* or *M. franciscanus*, but significantly increased with age in *L. variegatus* (1.9‐fold, *P* = 0.03, Fig. S5). Vasa immunostaining performed on coelomocytes from BrdU‐labeled animals indicated that Vasa and BrdU did not colocalize to the same cells (Fig. S6).

Immunohistochemistry was conducted on tube feet and spines from *L. variegatus* and *S. purpuratus* (*n* = 3 for each). In *L. variegatus*, Vasa was detected in cells of the epidermis along the stalk and disc of tube feet with little staining in the myoepithelium tissue that lines the lumen (Fig. 6). The amount of Vasa‐positive cells was estimated to be 4.42 ± 0.34%. The amount of Vasa staining in tube feet from *S. purpuratus* was difficult to quantitate due to background fluorescence of the connective tissue layer in this species (Fig. S4). Spines of *L. variegatus* and *S. purpuratus* show Vasa in cells throughout the dermis (Figs. 6 and S4). The Vasa‐positive cells were distributed along the length of the spines with 4.91 ± 0.49% in *L. variegatus* and 1.31 ± 0.16% in *S. purpuratus*. Immunohistochemistry on tube feet and spines from BrdU‐labeled *S. purpuratus* (*n* = 3) indicated that the Vasa‐positive cells were distinct from the cells that had actively incorporated BrdU in the 24 h labeling period (Fig. S7). The amount of BrdU incorporation for *S. purpuratus* spines was 2.37 ± 0.86% and tube feet 1.14 ± 0.43%. The dividing cells were distributed along the length of the tube feet, but for the spines there was more BrdU incorporation in cells near the base with less toward the tip.

## Discussion

Sea urchins exhibit continued growth and reproduction throughout their lives and yet different species are reported to have vastly different life expectancies in the wild. The proper balance of cell division and cell death is important for life‐long growth and homeostasis and maintaining this balance with age would be essential to achieve negligible senescence, whereas failure to maintain this balance would promote aging and shortened lifespan. In this study, it was determined that there was a low level of BrdU incorporation and apoptosis in the internal tissues (muscle, nerve, esophagus and coelomocytes) of three species of sea urchins with different lifespans, regardless of age. The low levels of cell proliferation are consistent with the low metabolic rates that have been reported for sea urchins (Ulbricht & Pritchard, 1972), and suggest that unlike *Hydra* (Schaible *et al*., 2014), sea urchins do not avoid senescence by continually replenishing tissues at a high rate. As expected for animals that grow indeterminately, there were higher levels of cell proliferation compared to apoptosis in the tissues. The low levels of apoptosis in the tissues of young and old animals are consistent with low levels of cellular damage that does not increase with age (Du *et al*., 2013). Interestingly, in *L. variegatus* and *S. purpuratus*, which have a ~ 10‐fold difference in lifespan, there were few differences in the amount of cell proliferation and apoptosis in tissues compared within age categories. The age range of *M. franciscanus* collected for this experiment was limited, so further investigation is required before a conclusion can be made for this species regarding changes with advanced age.

A decrease in the amount of BrdU incorporation was observed with increasing size/age, consistent with the slower growth of older animals (Ebert, 2007). However, this decrease may also be due to limited diffusion/distribution of BrdU into the tissues of larger animals during the 24 h labeling period. In support of this, a decrease in BrdU‐positive cells was observed in tissues of large *M. franciscanus* that are not that old with respect to their maximum lifespan potential. In addition, the expression of mRNA for genes associated with cell proliferation (*pcna*) and telomere maintenance (*tert*) was maintained with age in most tissues examined. The levels of BrdU incorporation in normal tissue homeostasis were similar to those observed in other marine animals such as the intestine of the sea cucumber, *Holothuria glabberrima* and tissues of the long‐lived clam *Arctica islandica* (< 1% BrdU‐positive nuclei in 24 h) (García‐Arrarás *et al*., 1998; Strahl & Abele, 2010). The level of BrdU incorporation in *A. islandica* was maintained with age in gill, mantle and adductor muscle, and only decreased significantly with age in heart tissue (Strahl & Abele, 2010). Contrary to the observation that tissues of sea urchin species with different lifespans show similar levels of BrdU incorporation, Strahl & Abele (2010) found a difference in bivalves with different lifespans, with high proliferation rates (> 10% BrdU‐positive nuclei) in tissues of a short‐lived scallop *Aequipecten opercularis*. However, these bivalves differ not only in their lifespans but also their life styles, with sedentary *A. islandica* burrowing beneath the sediment and extending a siphon into the water above for oxygen uptake and feeding, while *A. opercularis* is an actively swimming epibenthic scallop. Although the sea urchins used in this study come from different geographic locations, they are all herbivorous grazers collected in near‐shore waters (2–18 m depth).

To determine whether regenerative potential was maintained with age in sea urchins, the regrowth of amputated spines and tube feet was measured in *L. variegatus* and *S. purpuratus*. The data demonstrate that the regenerative potential of both types of appendages was maintained with age in these sea urchin species. A prediction from the evolutionary theories of aging is that level of extrinsic mortality influences the rate of aging, such that high levels of extrinsic mortality would be associated with tissue decline once an organism reaches reproductive maturity and survivorship in the wild becomes increasingly unlikely (Kirkwood, 2005; Kirkwood & Melov, 2011). *L. variegatus* is predicted to have a much lower annual survival rate than *S. purpuratus* and *M. franciscanus* (Ebert, 2007) and an estimated lifespan in the wild of about 4 years, and yet the results did not show evidence of decline in regenerative capacity in larger/older animals. It is possible that *L. variegatus* have the potential to live much longer than has been reported in the wild (and hence that the animals used in this study were not approaching their maximum lifespan potential), or that longevity in these animals is not limited by investment in (or capacity for) maintenance and repair of damaged tissues.

The widespread expression of *seawi* and *vasa* mRNA and presence of cells containing the Vasa protein in spines and tube feet as well as other somatic tissues (nerve, esophagus and a subpopulation of coelomocytes) suggest that multipotent cells may be important for normal tissue homeostasis in addition to regeneration of external appendages. The Vasa protein is posttranslationally regulated by targeted degradation in sea urchin embryos (Gustafson *et al*., 2011) which may explain why *vasa* mRNA is found in adult muscle tissue, yet no immunostaining of Vasa protein was observed in this tissue. This could mean that muscle does not have resident Vasa‐positive cells, or simply that they are located in regions that were not sampled. Aristotle's lantern musculature is composed of ten different smooth muscle groups (Ziegler *et al*., 2012) and only the interpyramidal muscles were sampled in this study.

A recent study reveals Vasa protein to be present in tissues of the sea urchin larvae and the adult rudiment, and its expression increases in response to tissue damage (Yajima & Wessel, 2015). The Vasa‐positive cells in the adult rudiment and larvae have higher BrdU incorporation than Vasa‐null cells, and embryos that lack Vasa were much less capable of repair of damage and eventually ceased development (Yajima & Wessel, 2015). Yajima & Wessel (2015) speculate that the widespread presence of Vasa in sea urchins may reflect the vast regulative and regenerative capabilities of embryos, larvae and adults. In support of this, the current study finds widespread presence of Vasa protein in somatic tissues of adult sea urchins. One notable difference is that Vasa‐positive cells in the larvae are highly proliferative (Yajima & Wessel, 2015), whereas in adult tissues in the current study BrdU‐labeled cells appear distinct from Vasa‐positive cells. The Vasa‐positive cells of adult sea urchin tissues may be cycling slowly or quiescent like the small micromeres of embryos, the Vasa‐expressing progenitors of adult tissues (Yajima & Wessel, 2015). Although the presence of Vasa protein and *seawi* mRNA is suggestive of stem cells, definitive proof requires demonstration that these cells have self‐renewal capacity and the capability to differentiate into different cell types.

The lack of age‐related differences in the maintenance of tissue homeostasis and regenerative potential in sea urchin species with different lifespans were unexpected in light of current evolutionary theories of aging, and further study is required to understand the factors underlying the short lifespan of *L. variegatus* in particular. Studies of the relationship between external mortality and senescence in guppies found that not all characteristics behaved as predicted by current theories for the evolution of senescence, indicating that the relationship is more complex than is currently understood (Reznick *et al*., 2004). Further studies of the intrinsic determinants of longevity in sea urchins will inform evolutionary theory as well as strategies for maintaining health with advancing age.

## Experimental procedures

### Collection of sea urchins and age determination

For cell turnover, gene expression, immunohistochemistry, and immunocytochemistry, *L. variegatus* were collected from Flatt's Inlet, Bermuda (32°19.4′N, 64°44.55′W) in January, 2013 under a special permit (SP130101) from the Bermuda Government Department of Environmental Protection, and *S. purpuratus* were collected in Mission Bay, San Diego, California (32°46.833′N, 117°14.557′W) in April, 2013. For cell turnover and immunohistochemistry, *M. franciscanus* were collected off San Diego (32°51.26′N 117°16.89′W) in April, 2013. *M. franciscanus* used for gene expression and coelomocyte immunocytochemistry were collected near Kendrick Island in Gabriola Pass, British Columbia (49°07.554′N, 123°41.461′W) in August 2013. For tissue regeneration experiments, *L. variegatus* were collected in Mangrove Bay Bermuda (32°22.272′N, 64°41.650′W) in April 2014 and *S. purpuratus* were collected off San Diego California (32°39.954′N, 117°15.759′W) in June 2015. Following collection, *S. purpuratus* used for the tissue regeneration experiment were kept in recirculating sea water, and all other sea urchins were kept in flow‐through aquaria. Animals were fed *ad libitum*:* L. variegatus* were given a mixture of sea grass (*Thalassia testudinum*), collected under a Protected Species License granted by the Government of Bermuda Department of Conservation Services, and algae from their natural environment. *S. purpuratus* and *M. franciscanus* were fed giant kelp (macrocystis) collected from their natural environment. BrdU incubations and tissue dissections were conducted within 1 week of collections.

*Lytechinus variegatus* ages were estimated using test (shell) diameter and comparisons with previously established growth curves generated from size frequency and growth band counting data collected in Florida and Bermuda (Moore *et al*., 1963; Beddingfield & McClintock, 2000). *S. purpuratus* ages were estimated from test diameter using growth curves previously generated from the Tanaka parameters from tetracycline tagging experiments conducted at Mission Bay, San Diego (*f* = 1.30757, *d* = 2.80184, *a* = 0.16314) (Coleman, 1993). *M. franciscanus* age estimates were based on growth curves generated from tetracycline tagged sea urchins in Oregon and Washington using the Tanaka function (*f* = 0.22929, *d* = 6.07531, *a* = 0.19906) (Ebert, 1998, 2008; Ebert *et al*., 1999).

### Analysis of cell proliferation

Cell proliferation was measured using *in vivo* incorporation of 5‐bromo‐2′‐deoxyuridine (BrdU). Twenty‐four hours following injection of BrdU into the coelomic cavity (5 mg BrdU per 100 g body weight), tissues and cells were preserved in 4% paraformaldehyde and tissues were embedded in paraffin, sectioned (5 μm). Tissues were deparaffinized with toluene, rehydrated, unmasked with 10 mm sodium citrate solution (pH 6.0) and cells and tissues were permeablized in methanol. DNA was denatured and neutralized prior to blocking for 1 h in 4% BSA in PBST (PBS with 0.1% Tween 20), followed by an overnight incubation at 4 °C with anti‐BrdU conjugated to Alexa Fluor^®^ 488 (1:100 dilution in 2% BSA in PBST) (Life Technologies, Carlsbad, CA, USA). After washing with PBST, cells and tissues were mounted with Citifluor^™^ containing DAPI (1.67 μg mL^−1^). Negative controls were cells and tissue sections from sea urchins that were not BrdU‐labeled that were developed simultaneously with the BrdU‐labeled samples.

For analysis, 12 nonoverlapping fields for each tissue were randomly selected using an Olympus AX70 epifluorescence microscope (Olympus, Tokyo, Japan) and images captured in each fluorescent channel with a Retiga EXi Digital camera (Qimaging, Surrey, BC, Canada). The BrdU‐positive nuclei were quantified as a percentage of total number of nuclei using Image Pro Plus version 7.0 software (Media Cybernetics, Bethesda, MD, USA) and values for all 12 fields were averaged for each tissue section. BrdU incorporation (%) data were arcsine transformed and species‐ and age‐specific differences were analyzed using the general linear model for comparison within each tissue type (Statgraphics Centurion; Statpoint Technologies, Inc., Warrenton, VA, USA). Comparisons between the young and old groups within a tissue were conducted using *t*‐tests for equal or unequal variances if normally distributed, or using the Mann−Whitney test if the data were not normally distributed. Species‐specific differences were evaluated using one‐way ANOVA for normally distributed data and the Kruskal−Wallis test if the data were not normally distributed, followed by multiple range *post hoc* tests (Statgraphics Centurion).

### Analysis of apoptosis

Apoptosis was assessed using Apo ssDNA, an antibody based assay to detect single‐stranded DNA in apoptotic cells (Cell Technology, Fremont, CA, USA). The protocol provided with the Apo ssDNA kit was followed to develop the slides containing paraformaldehyde‐fixed, paraffin embedded sea urchin tissue sections or the protocol for cell suspension for coelomocytes. Following partial denaturation in 50% formamide and washing, a 1:10 dilution of ssDNA monoclonal antibody (F7‐26) was used with a 1:100 dilution of the FITC‐conjugated anti‐mouse IgM secondary antibody. For the negative control samples, the Apo ssDNA primary antibody was omitted from the procedure.

Apoptosis was also evaluated on tissue sections using the APO‐BrdU^™^ TUNEL assay kit (Life Technologies). Slides containing paraformaldehyde‐fixed, paraffin embedded tissue sections were deparaffinized with toluene, rehydrated, unmasked with 10 mm sodium citrate (pH 6.0) and permeablized in methanol. The DNA‐labeling reaction was conducted at 37 °C for 4 h followed by 12 h at room temperature. The Alexa Fluor^®^ 488 conjugated anti‐BrdU antibody was used at a 1:50 dilution. For the negative controls, terminal deoxynucleotide transferase was omitted from the labeling reaction.

For both apoptosis assays, the tissues were mounted with Citifluor^™^ containing DAPI (1.67 μg mL^−1^) and image collection and analysis were conducted as described above for cell proliferation (BrdU incorporation).

### Preparation of RNA and qRT‐PCR

Dissected sea urchin tissues were preserved in RNA later solution (Qiagen, Valencia, CA, USA), coelomocytes were pelleted by centrifugation at 6000 *g* for 5 min and all samples were stored at −80 °C. RNA was extracted using the Trizol reagent (Invitrogen/Life Technologies, Carlsbad, CA, USA) followed by the RNA clean‐up protocol of the RNeasy mini Kit (Qiagen). Differential expression of selected genes was examined using qRT‐PCR on the ABI 7300 Real Time‐PCR machine using the SYBR Green detection system (Applied Biosystems, Foster City, CA, USA). RNA was reverse‐transcribed using the High Capacity cDNA Reverse Transcription Kit (Applied Biosystems) and primers for qRT‐PCR were designed using Primer Express software (version 3.0) (Applied Biosystems) based on sequences of target genes identified using the echinoderm genome database (http://www.echinobase.org). Primer sequences are listed in Table S3. The expression level of genes was determined using the relative quantification method described by Hellemans *et al*. (2007) corrected for gene‐specific amplification efficiencies and normalized to a factor derived from the geometric mean of three stably expressed control genes determined by qbase^+^ software (Biogazelle, Zwijnaarde, Belgium). For each gene within each tissue type, the sample with the highest expression was used as the calibrator or reference sample, and the values for the different groups (young vs. old) were averaged.

### Immunohistochemistry/immunocytochemistry

Tissues and cells were fixed in 4% paraformaldehyde in PBS. Spines were decalcified by overnight incubation in Immunocal^™^ and all tissues were embedded in paraffin and sectioned (5 μm). Tissues and cells were treated with the anti‐Vasa antibody [1 μg mL^−1^ anti‐Vasa, Developmental Studies Hybridoma Bank (DSHB)] and imaged as previously described (Reinardy *et al*., 2015).

The DSHB anti‐Vasa antibody used in this study was produced to *D. melanogaster* Vasa, but has been previously shown to detect sea urchin Vasa in the small micromeres of *S. purpuratus* embryos (Campanale *et al*., 2014). The antibody was raised to amino acids 16–433 of *D. melanogaster* Vasa and an alignment of this region with *L. variegatus* and *S. purpuratus* Vasa shows good sequence conservation across the entire region with stronger conservation for amino acids 202–433 (46.6% identity and 66.8% similarity for *L. variegatus*, and 51.0% identity and 71.1% similarity for *S. purpuratus*) (Fig. S8). The specificity of this antibody was confirmed using an affinity‐purified antibody produced to the DEAD‐box region of *S. purpuratus* Vasa (Voronina *et al*., 2008). A double‐labeling experiment using this antibody together with the *D. melanogaster* Vasa antibody gave overlapping immunostaining patterns (Fig. S9).

### Tube feet and spine regeneration assay

Tissue regeneration was measured using an assay that evaluates the regrowth of amputated tube feet and spines every 7 days over a 5‐week period as previously described (Reinardy *et al*., 2015). Tube feet and spines (10% of total) were removed from one of the ambulacral segments of the test from the oral to aboral surface (Fig. 1B–D). At each time point, ten regenerating spines and tube feet were measured along the cut area and ten full‐length spines and tube feet were measured in the adjacent area. The regrowth data, expressed as percentages of full‐length spines and tube feet, were arcsine transformed and used to determine time‐ and age‐specific differences using the general linear model and *post hoc* tests (Statgraphics Centurion, Statpoint Technologies, Inc., Warrenton, VA, USA). Comparisons between the young and old groups were conducted using t‐tests for equal or unequal variances for normally distributed data or using the Mann−Whitney test if the data were not normally distributed.

## Author contributions

AGB conceived and designed the experiments, participated in the acquisition and analysis of data and wrote the manuscript. JAC participated in the design and/or acquisition of data for cell proliferation, apoptosis, immunohistochemistry of tube feet and spines, and edited the manuscript.

## Funding

This work was supported by Grant R21AG039761 from the National Institute on Aging. Additional financial support was provided by a Bermuda Charitable Trust and the Christian Humann Foundation, the Salisbury Cove Research Fund at MDI Biological Laboratory, and Institutional Development Awards (IDeA) to the MDI Biological Laboratory from the NIGMS of the NIH, under grant numbers P20‐GM104318 and P20‐GM103423.

## Conflict of interest

The authors have no conflict of interest to declare.

## Supporting information


**Fig. S1** Total number of cells counted and images for BrdU analysis of sea urchin tissues.

**Fig. S2** Total number of cells counted and images for TUNEL analysis of sea urchin tissues.

**Fig. S3** Total number of cells counted and images for Apo ssDNA analysis of sea urchin tissues.

**Fig. S4** Immunohistochemistry and immunocytochemistry of sea urchin tissues and cells using an antibody to Vasa.

**Fig. S5** Percent of Vasa‐positive coelomocytes from *Lytechinus variegatus* (Lv), *Strongylocentrotus purpuratus* (Sp), and *Mesocentrotus franciscanus* (Mf).

**Fig. S6** Immunocytochemistry of coelomocytes from BrdU‐treated *Lytechinus variegatus* (Lv), *Strongylocentrotus purpuratus* (Sp), and *Mesocentrotus franciscanus* (Mf) developed with anti‐BrdU and anti‐Vasa antibodies.

**Fig. S7** Immunohistochemistry of *Strongylocentrotus purpuratus* spines showing BrdU‐positive nuclei are distinct from Vasa‐positive cells.

**Fig. S8** Clustal W2: clustal 2.1 multiple sequence alignment of Vasa from *Drosophila melanogaster*,* Lytechinus variegatus* and *Strongylocentrotus purpuratus*.

**Fig. S9** Immunohistochemistry double‐labeling experiment on *Lytechinus variegatus* spines using anti‐*Strongylocentrotus purpuratus* Vasa and anti‐*Drosophila melanogaster* Vasa.

**Table S1** Average threshold cycle (Ct) values from qRT‐PCR for cell proliferation, multipotency and control genes in sea urchin tissues.

**Table S2** Expression of *nanos2* in tissues of *Lytechinus variegatus* (Lv), *Strongylocentrotus purpuratus* (Sp), and *Mesocentrotus franciscanus* (Mf).

**Table S3** qRT‐PCR primer sequences.
