# Supplementary material for: Maintenance of somatic tissue regeneration with age in short‐ and long‐lived species of sea urchins
Source: Aging Cell. 2016 Apr 20;15(4):778–87. doi: 10.1111/acel.12487 (PMC4933669; doi:10.1111/acel.12487)
Supplement: Supplementary file 1 — Fig. S1 Total number of cells counted and images for BrdU analysis of sea urchin tissues. [file ACEL-15-778-s001.pdf]

Total number of cells counted for BrdU analysis of sea urchin tissues

BrdU	ALM		ES		RN		Coel	
	**Young**	**Old**	**Young**	**Old**	**Young**	**Old**	**Young**	**Old**
Lv	49589	37623	103294	98237	102649	121622	23015	10950
Sp	36747	35335	94903	86915	114736	90319	13589	14048
Mf	45891	37958	94724	103877	105334	72652	7073	9084

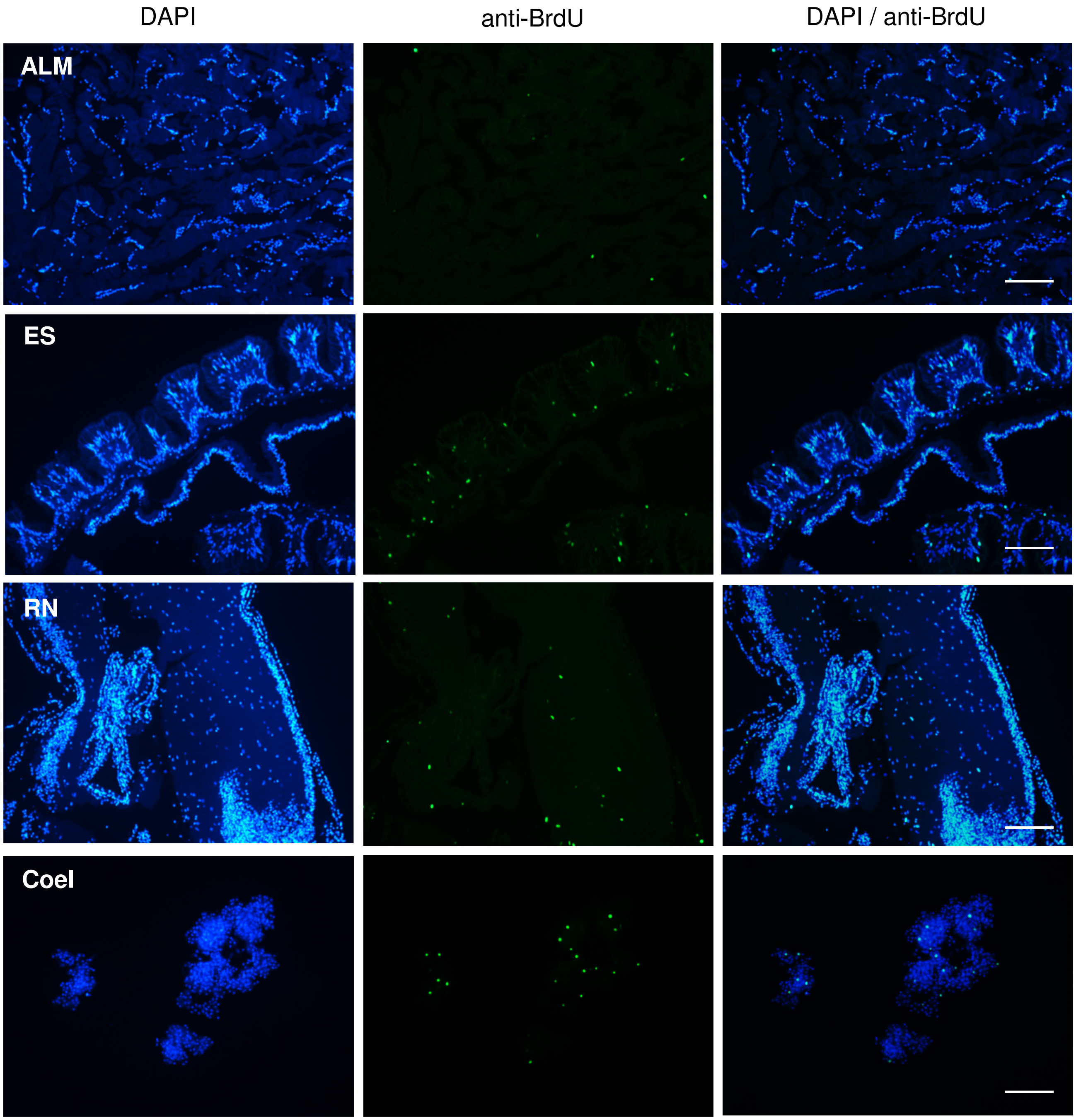

Fig. S1 Total number of cells counted and images for BrdU analysis of sea urchin tissues. Selection of images from *L. variegatus* tissues [Aristotle's lantern muscle (ALM), esophagus (ES), radial nerve (RN) and coelomocytes (Coel)] labeled with anti-BrdU or stained with DAPI. Scale bar represents 100 µm.
