# Supplementary material for: Maintenance of somatic tissue regeneration with age in short‐ and long‐lived species of sea urchins
Source: Aging Cell. 2016 Apr 20;15(4):778–87. doi: 10.1111/acel.12487 (PMC4933669; doi:10.1111/acel.12487)
Supplement: Supplementary file 2 — Fig. S2 Total number of cells counted and images for TUNEL analysis of sea urchin tissues. [file ACEL-15-778-s002.pdf]

Total number of cells counted for TUNEL analysis of sea urchin tissues

TUNEL	ALM		ES		RN	
	Young	Old	Young	Old	Young	Old
Lv	46612	43969	89762	85889	108782	90941
Sp	44768	47431	82109	80411	113574	68783
Mf	45687	37714	84201	95374	110220	73316

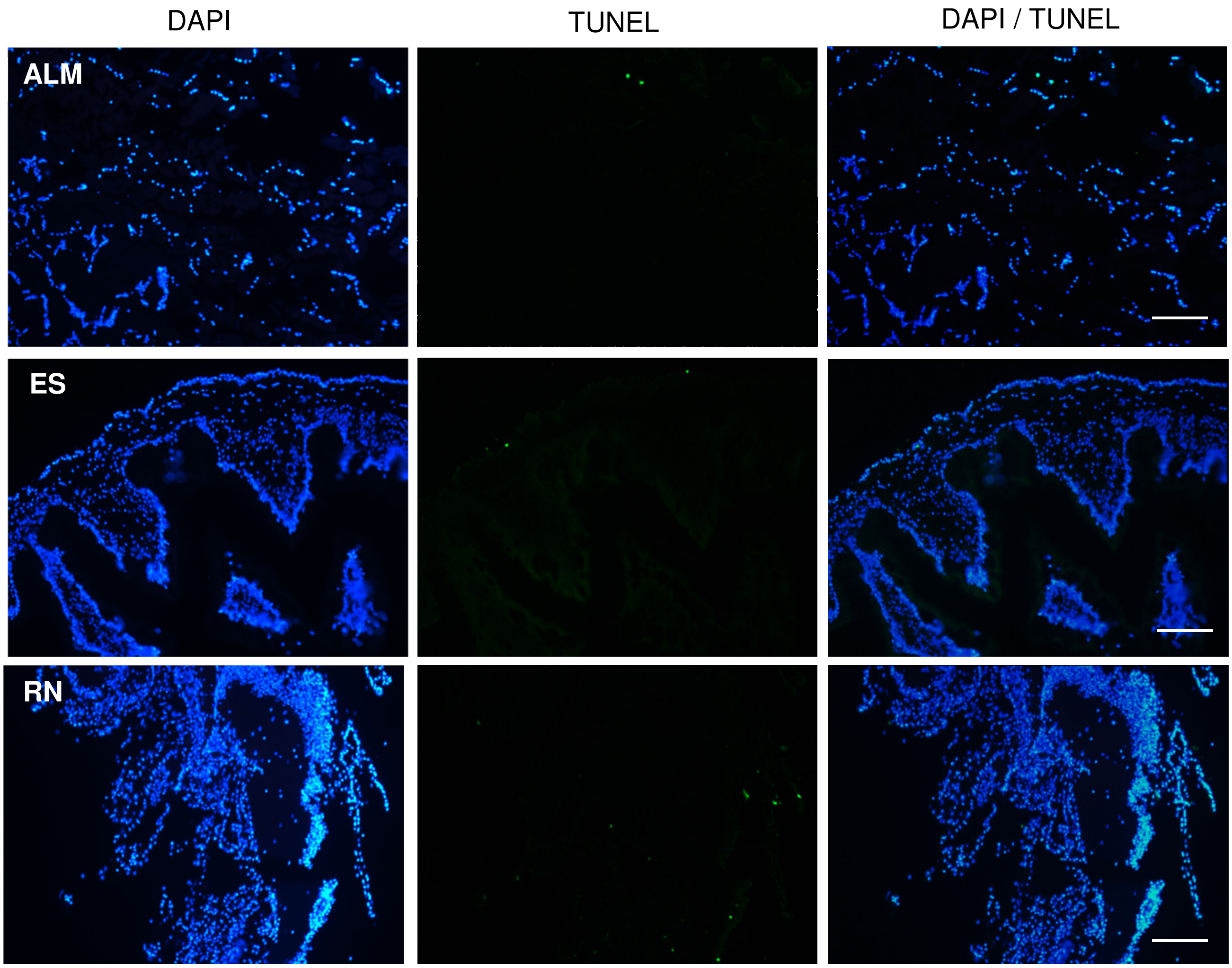

Fig. S2 Total number of cells counted and images for TUNEL analysis of sea urchin tissues. Selection of images from *L. variegatus* tissues [Aristotle's lantern muscle (ALM), esophagus (ES), radial nerve (RN)] developed using the TUNEL assay and stained with DAPI. Scale bar represents 100 µm.
