# Supplementary material for: Maintenance of somatic tissue regeneration with age in short‐ and long‐lived species of sea urchins
Source: Aging Cell. 2016 Apr 20;15(4):778–87. doi: 10.1111/acel.12487 (PMC4933669; doi:10.1111/acel.12487)
Supplement: Supplementary file 3 — Fig. S3 Total number of cells counted and images for Apo ssDNA analysis of sea urchin tissues. [file ACEL-15-778-s003.pdf]

Total number of cells counted for Apo ssDNA analysis of sea urchin tissues

ApossDNA	ALM		ES		RN		Coel	
	**Young**	**Old**	**Young**	**Old**	**Young**	**Old**	**Young**	**Old**
Lv	21276	19252	52411	53127	57974	52915	55246	36775
Sp	22547	20553	43447	42757	46531	41968	24730	14902
Mf	22467	21899	56367	57394	43720	30597	20044	20486

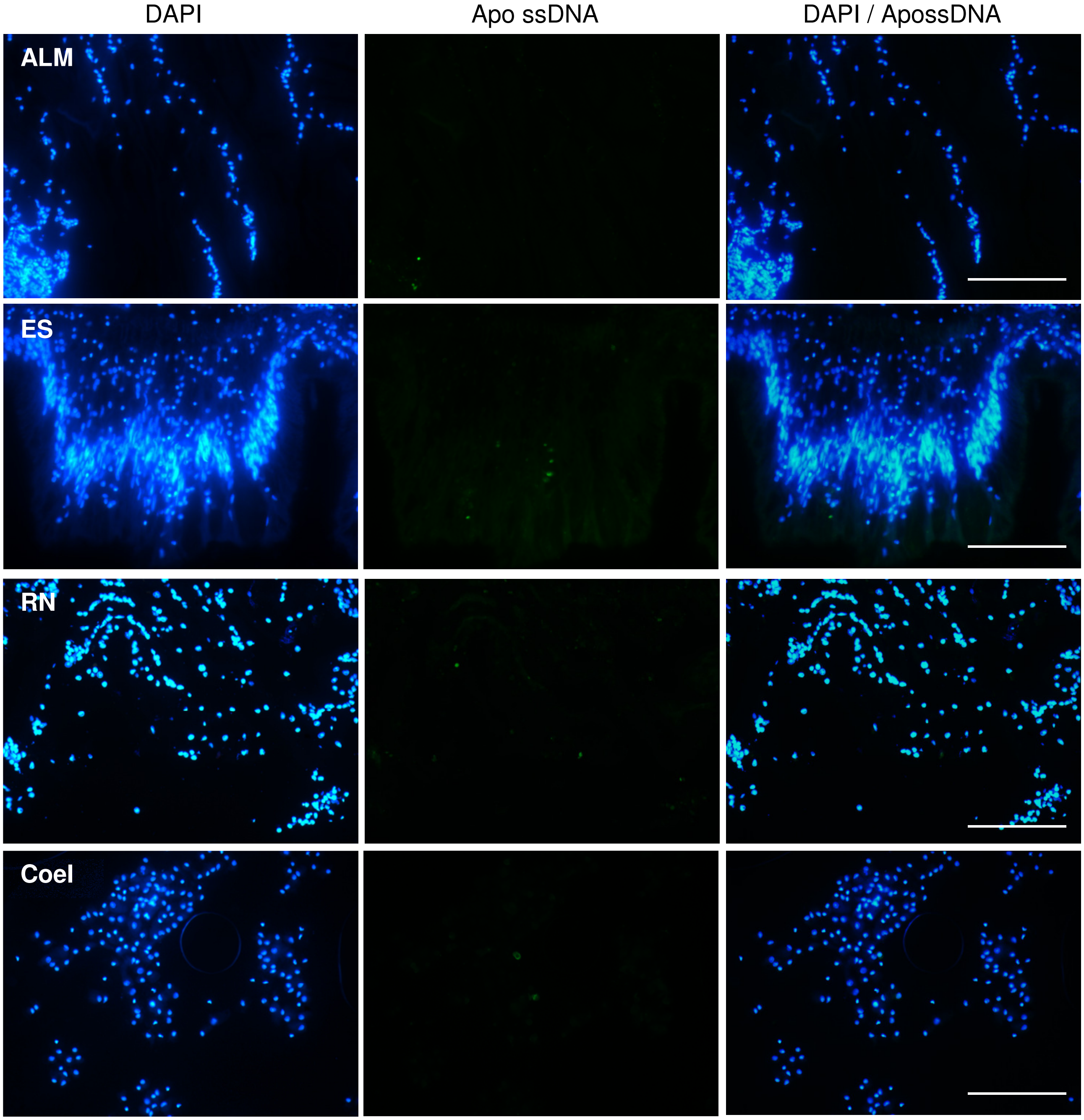

Fig. S3 Total number of cells counted and images for Apo ssDNA analysis of sea urchin tissues. Selection of images from *L. variegatus* tissues [Aristotle's lantern muscle (ALM), esophagus (ES), radial nerve (RN) and coelomocytes (Coel)] developed using the Apo ssDNA assay and stained with DAPI. Scale bar represents 100 μm.
