# Supplementary material for: Maintenance of somatic tissue regeneration with age in short‐ and long‐lived species of sea urchins
Source: Aging Cell. 2016 Apr 20;15(4):778–87. doi: 10.1111/acel.12487 (PMC4933669; doi:10.1111/acel.12487)
Supplement: Supplementary file 4 — Fig. S4 Immunohistochemistry and immunocytochemistry of sea urchin tissues and cells using an antibody to Vasa. [file ACEL-15-778-s004.pdf]

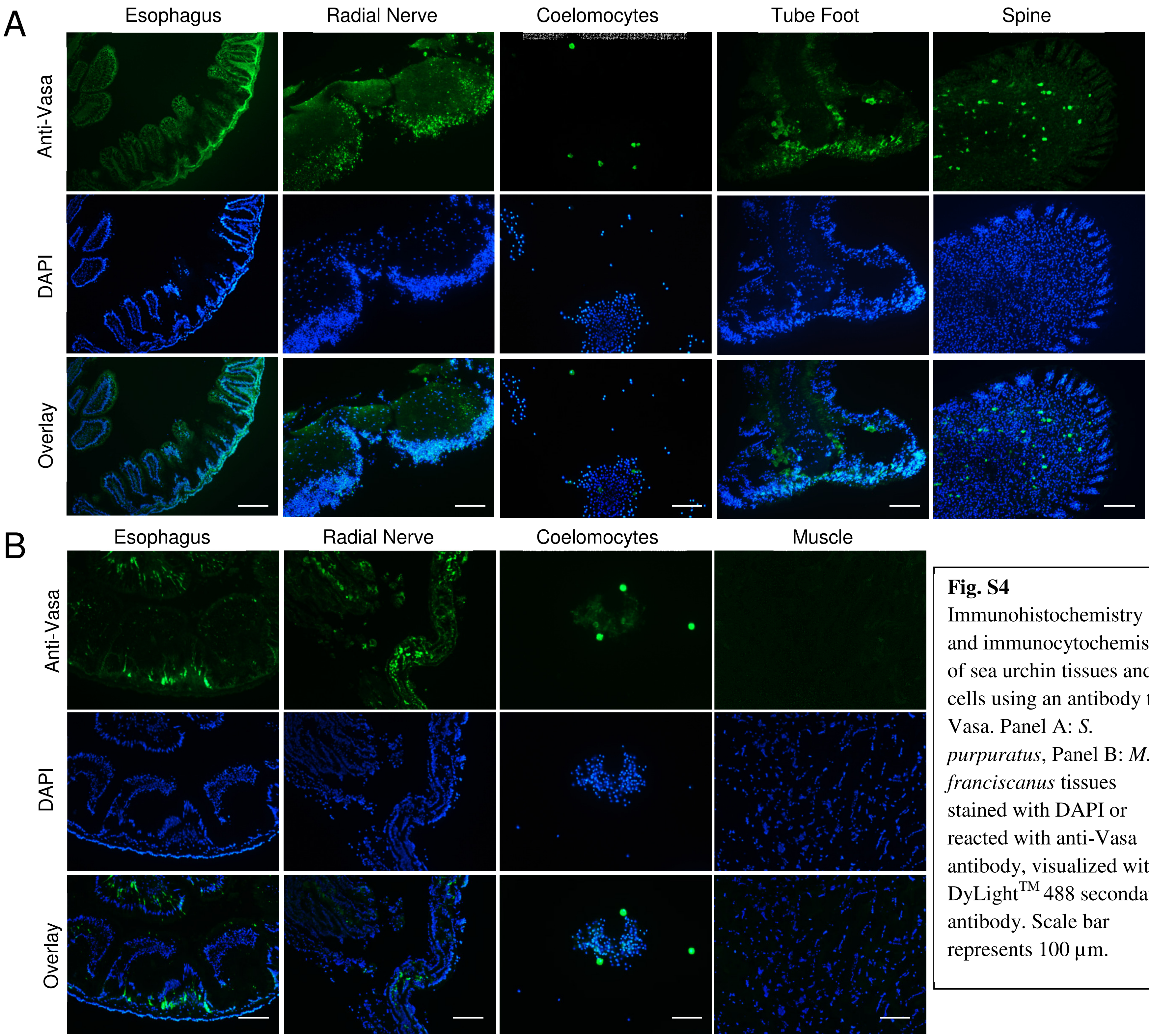

Fig. S4 Immunohistochemistry and immunocytochemistry of sea urchin tissues and cells using an antibody to Vasa. Panel A: *S. purpuratus*, Panel B: *M. franciscanus* tissues stained with DAPI or reacted with anti-Vasa antibody, visualized with DyLight^{TM} 488 secondary antibody. Scale bar represents 100 µm.
