# Supplementary material for: Maintenance of somatic tissue regeneration with age in short‐ and long‐lived species of sea urchins
Source: Aging Cell. 2016 Apr 20;15(4):778–87. doi: 10.1111/acel.12487 (PMC4933669; doi:10.1111/acel.12487)
Supplement: Supplementary file 5 — Fig. S5 Percent of Vasa‐positive coelomocytes from Lytechinus variegatus (Lv), Strongylocentrotus purpuratus (Sp), and Mesocentrotus franciscanus (Mf). [file ACEL-15-778-s005.pdf]

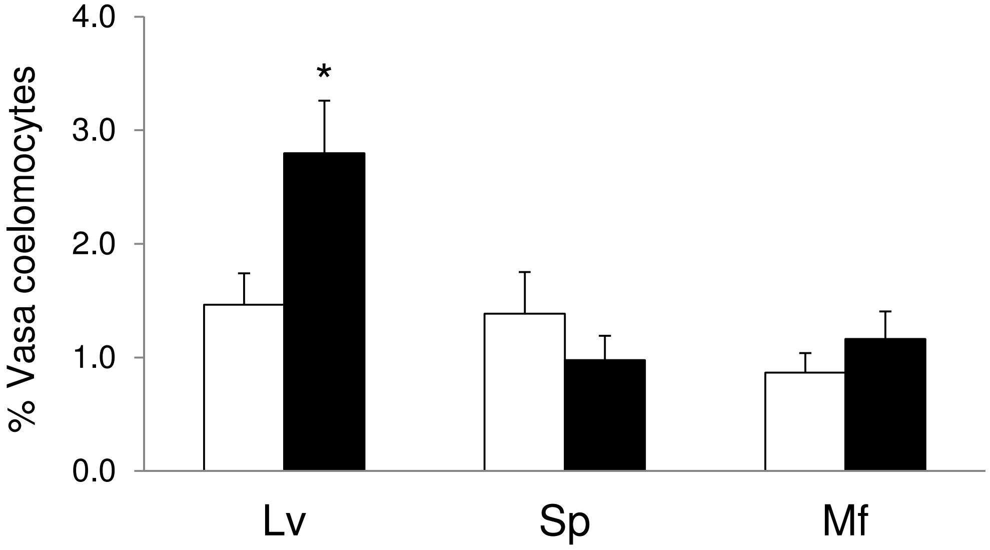

Fig. S5 Percent of Vasa-positive coelomocytes from *L. variegatus* (Lv), *S. purpuratus* (Sp) and *M. franciscanus* (Mf). The white bars represent small/young animals and the black bars represent large/old animals. Age estimates and number of animals in each group are shown in Table 1. The data are presented as means and standard errors, and the star (*) indicates a significant difference between age groups ($p<0.05$).
