# Supplementary material for: Maintenance of somatic tissue regeneration with age in short‐ and long‐lived species of sea urchins
Source: Aging Cell. 2016 Apr 20;15(4):778–87. doi: 10.1111/acel.12487 (PMC4933669; doi:10.1111/acel.12487)
Supplement: Supplementary file 6 — Fig. S6 Immunocytochemistry of coelomocytes from BrdU‐treated Lytechinus variegatus (Lv), Strongylocentrotus purpuratus (Sp), and Mesocentrotus franciscanus (Mf) developed with anti‐BrdU and anti‐Vasa antibodies. [file ACEL-15-778-s006.pdf]

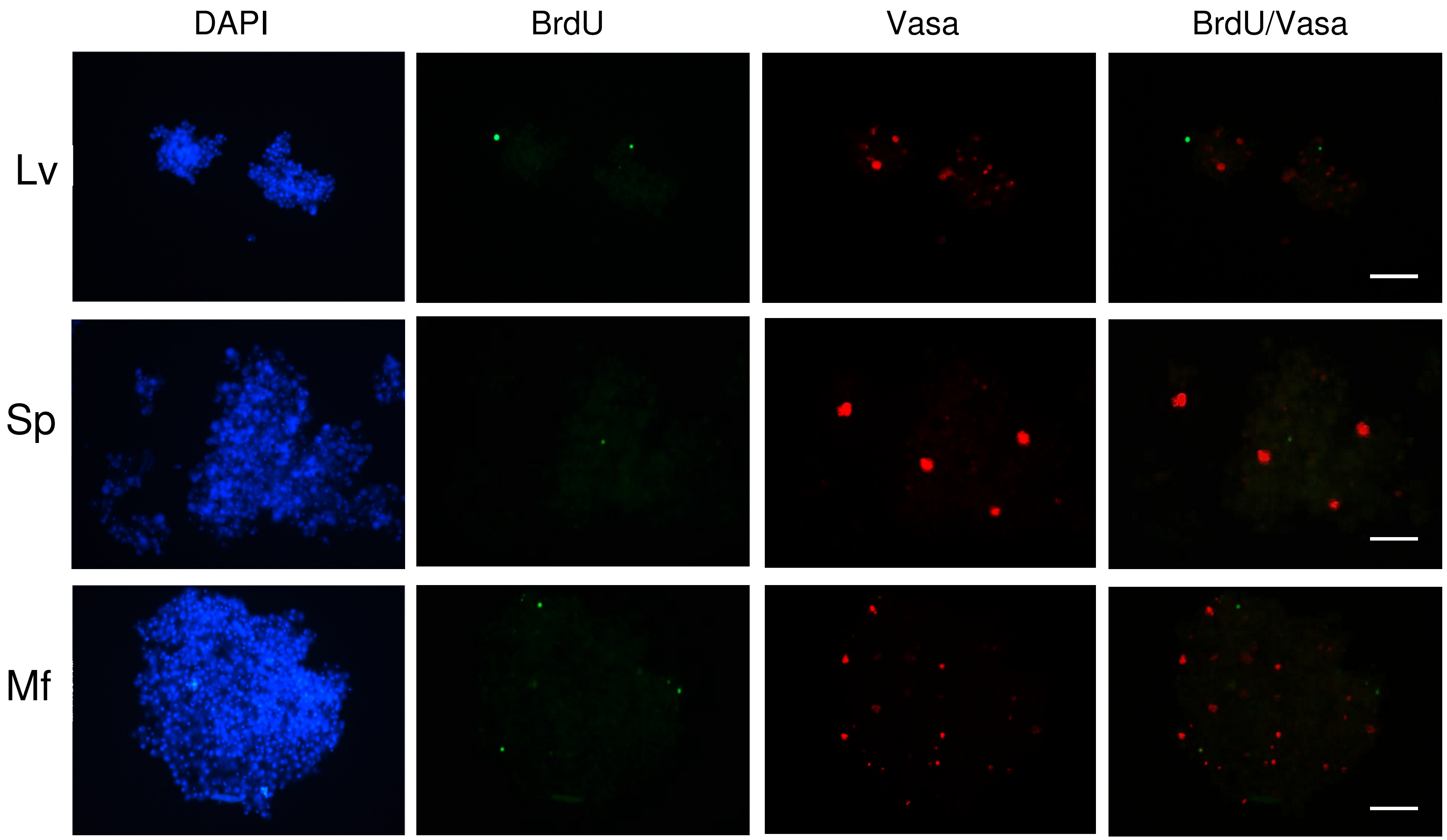

Fig. S6 Immunocytochemistry of coelomocytes from BrdU-treated *L. variegatus* (Lv), *S. purpuratus* (Sp) and *M. franciscanus* (Mf) developed with anti-BrdU and anti-Vasa antibodies: BrdU-incorporation (anti-BrdU conjugated to Alexa Fluor® 488), Vasa immunostaining (anti-Vasa was detected with a DyLight™647 conjugated secondary antibody) and DAPI shows all nuclei. Scale bar represents 100 µm.
