# Supplementary material for: Maintenance of somatic tissue regeneration with age in short‐ and long‐lived species of sea urchins
Source: Aging Cell. 2016 Apr 20;15(4):778–87. doi: 10.1111/acel.12487 (PMC4933669; doi:10.1111/acel.12487)
Supplement: Supplementary file 7 — Fig. S7 Immunohistochemistry of Strongylocentrotus purpuratus spines showing BrdU‐positive nuclei are distinct from Vasa‐positive cells. [file ACEL-15-778-s007.pdf]

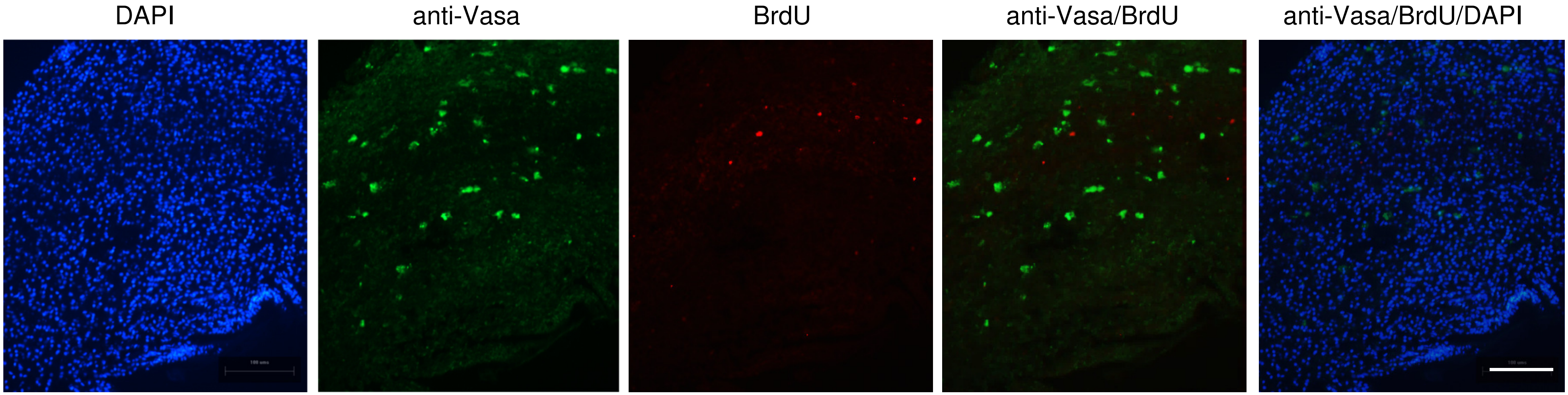

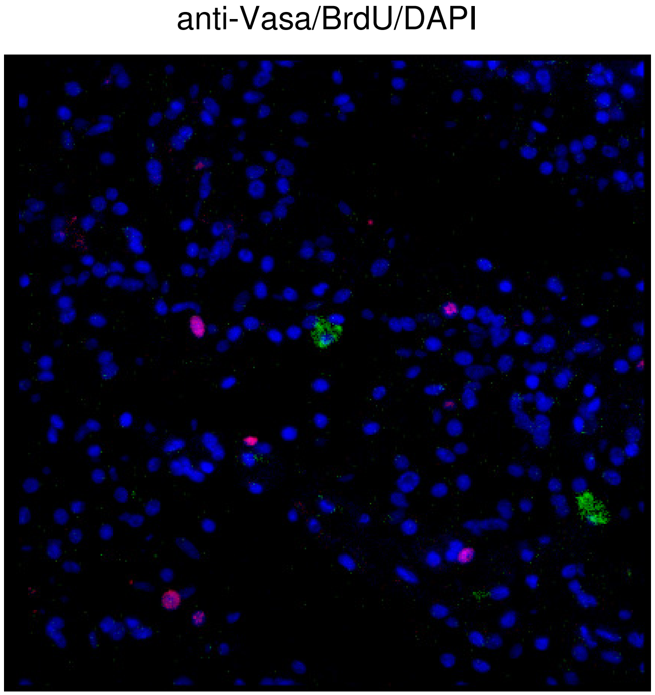

Fig. S7 Immunohistochemistry of *S. purpuratus* spines showing BrdU-positive nuclei are distinct from Vasa-positive cells. Scale bar represents 100 µm.
