# Supplementary material for: Maintenance of somatic tissue regeneration with age in short‐ and long‐lived species of sea urchins
Source: Aging Cell. 2016 Apr 20;15(4):778–87. doi: 10.1111/acel.12487 (PMC4933669; doi:10.1111/acel.12487)
Supplement: Supplementary file 8 — Fig. S8 Clustal W2: clustal 2.1 multiple sequence alignment of Vasa from Drosophila melanogaster, Lytechinus variegatus and Strongylocentrotus purpuratus. [file ACEL-15-778-s008.pdf]

Fig. S8 Clustal W2: CLUSTAL 2.1 multiple sequence alignment of Vasa from *D. melanogaster*, *L. variegatus* and *S. purpuratus*.

```
Vasa, isoform A [Drosophila melanogaster] GenBank: AAF53438 (amino acids 16-433)
Vasa [Lytechinus variegatus] GenBank: ACM80368 (amino acids 42-433)
Vasa [Strongylocentrotus purpuratus] GenBank: NM_001146193 or SPU_008908 Peptide
Sequence (amino acids 10-516)

Lv_Vasa         LRGGTSFEK----KSYGGSSSGFGSKANGFGIGVGRGRGRGRGFQSFAEQ--GGVGGLTN 95
Sp_Vasa         VSGGSSFGK----SSFGGSSG--GVKDDGFGVGVGRGRGRGRGFSPFASN--GGVGGMTN 61
Dm_Vasa         ARGGDWSDDEDTAKSFSGEAEGDGVGGSG-GEGGGYQGGNRDVFGRIGGGRGGGAGGYRG 74
                  **    .    .*:.*.:   *   .* * * *   *.   *  :.    **.**  .

Lv_Vasa         GTTNGTSNGGDSGWNDPPS-NGTSSSPWDDSSSSGAGKRSFESKGSFGG-GGGGGRGGR- 152
Sp_Vasa         GTTNGGGES-SGGWDESPSKSGSSSSPWGGDGDNGFGKSSFGSK-SFGGRGGGGGGGGRG 119
Dm_Vasa         GNRDGGGFHGGRREGERDFRGGEGGFRGGQGGSRGGQGGSRGGQGGFRG--GEGGFRGRL 132
                *. :* .   .   .:    .* ..   . ... *    *  .: .* *  * **  **

Lv_Vasa         -----DRGGRSFGDSENGGGG---------------------------------RSFX-- 172
Sp_Vasa         AESGGGRGGGGFGGRREGGGGGGGGGACYKCQEEGHMARDCPNGDSSGGGRGGGDRSCYNC 179
Dm_Vasa         YENEDGDERRGRLDREERGGE----------RRGRLDREERGGERGERGDGGFARRRRNE 182
                     .    .  . .: **                                  *

Lv_Vasa         ------------------------------------------------------------
Sp_Vasa         GETGHMSRECPTKDSSGGGRGGGGGDRSCYNCGETGHMSRECPTKDSSGGGGGGGGGKCFR 239
Dm_Vasa         DDIN-------------------------------------------------------- 186


Lv_Vasa         ----------------RPEGEGGEAKPPASTYIPPPPSEEEEQIYMS-TXQGINFNRYDD 215
Sp_Vasa         CQEEGHFAKECPNPDTRNEGEGGEAKPPASTYVPPDPSEEESQIYIS-TEQGINFNKYDD 298
Dm_Vasa         ----------------NNNNIVEDVERKREFYIPPEPSNDAIEIFSSGIASGIHFSKYNN 230
                                . :.   :.:   . *:** **::  :*: *   .**:*.:*::

Lv_Vasa         IPVEVSGRDGPKHIRSFEEAGLDETVLENVRKARYAKPTPVQKYAIPIIGAGLDLMACAQ 275
Sp_Vasa         IPVEVSGRDRPKNIRAFEEAELDETVLSNVRKAHYAKPTPVQKYGMPIISCGRDLMACAQ 358
Dm_Vasa         IPVKVTGSDVPQPIQHFTSADLRDIIIDNVNKSGYKIPTPIQKCSIPVISSGRDLMACAQ 290
                ***:*:* * *: *: * .* * : ::.**.*: *  ***:** .:*:*..* *******

Lv_Vasa         TGSGKTAAFLLPIITNMITQSGCVSCFSVVQEPLALIVSPTRELASQIQNEARKFCRNTS 335
Sp_Vasa         TGSGKTAAFLLPIITNMITHGGCISTFNVIQEPLALIVSPTRELAIQIYNEARKFCRGTT 418
Dm_Vasa         TGSGKTAAFLLPILSKLLEDP----HELELGRPQVVIVSPTRELAIQIFNEARKFAFESY 346
                *************::::: .         : .* .:********* ** ******.  :

Lv_Vasa         LRPVVIYGGTSVSHQTREVQNGCSILVATPGRMHDFIGRGYIGLGKLKYLILDEADRMVD 376
Sp_Vasa         IRPVVVYGGTSVSHQMRECQKGCSILVATPGRMHDFVTRGYIGLGKLKFLILDEADRMLD 478
Dm_Vasa         LKIGIVYGGTSFRHQNECITRGCHVVIATPGRLLDFVDRTFITFEDTRFVVLDEADRMLD 406
                ::  ::*****. ** .   .** :::*****: **: * :* : . ::::*******:*

Lv_Vasa         MGFGPEIQKLIDHPHMPPKGERQTLMFSATFPPEIQEK 433
Sp_Vasa         MGFGPDIQRLIEHPHMPAKGDRQTLMFSATFPNEVQEK 516
Dm_Vasa         MGFSEDMRRIMTHVTMRP--EHQTLMFSA--------- 433
                ***. :::::: *  * .  ::*******
```
