# Supplementary material for: Maintenance of somatic tissue regeneration with age in short‐ and long‐lived species of sea urchins
Source: Aging Cell. 2016 Apr 20;15(4):778–87. doi: 10.1111/acel.12487 (PMC4933669; doi:10.1111/acel.12487)
Supplement: Supplementary file 9 — Fig. S9 Immunohistochemistry double‐labeling experiment on Lytechinus variegatus spines using anti‐Strongylocentrotus purpuratus Vasa and anti‐Drosophila melanogaster Vasa. [file ACEL-15-778-s009.pdf]

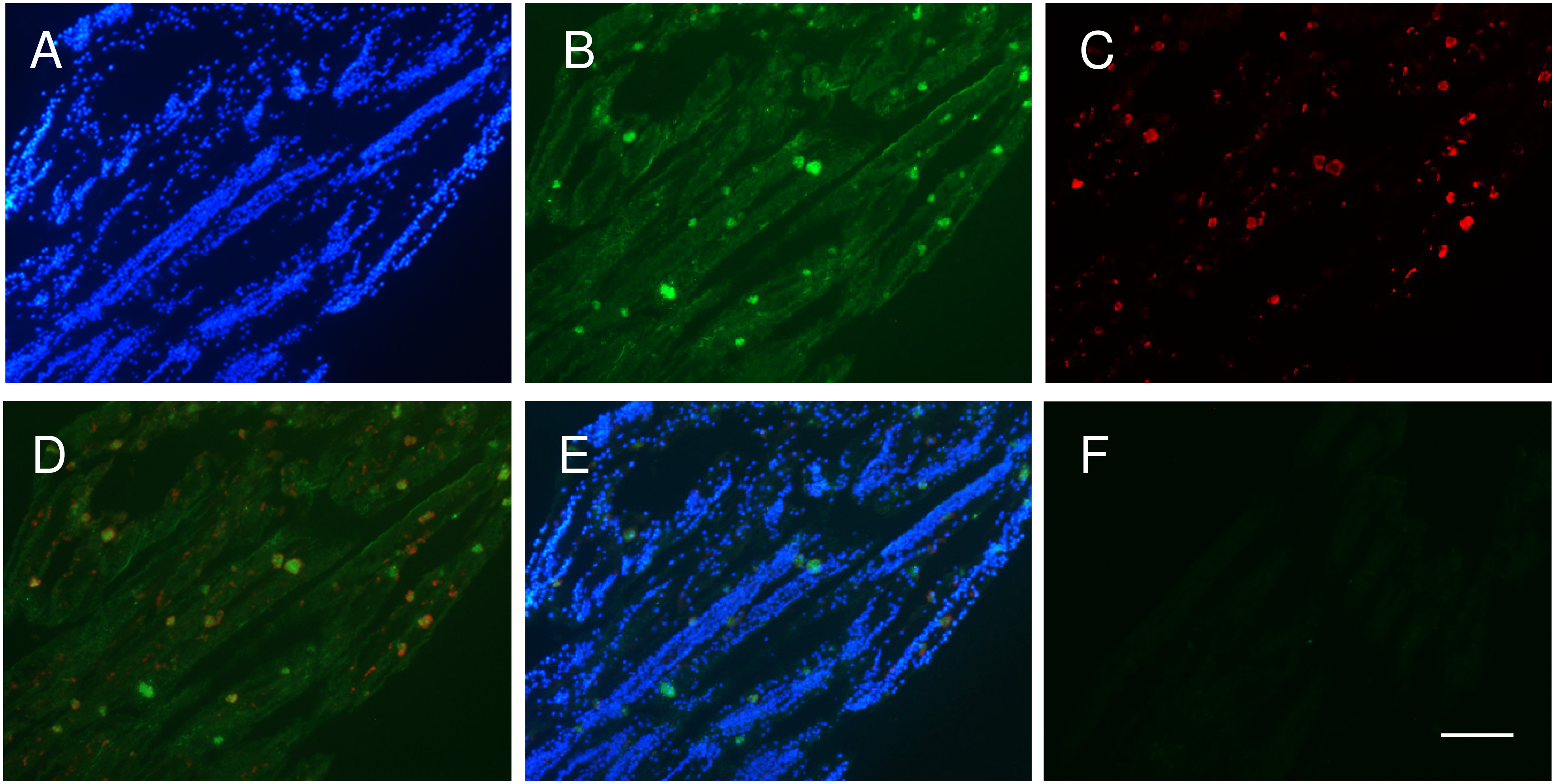

Fig. S9 Immunohistochemistry double-labeling experiment on *L. variegatus* spines using anti-*S. purpuratus* Vasa and anti-*D. melanogaster* Vasa. Panel A: DAPI image, Panel B: anti-*S. purpuratus* Vasa (Alexa Fluor® 488), Panel C: anti-*D. melanogaster* Vasa (DyLight™647), Panel D: overlay of image B and C, Panel E: overlay of image A, B and C, Panel F: overlay of the negative control images using secondary antibodies labeled with Alexa Fluor® 488 and DyLight™647, but omitting the primary antibodies to Vasa. The anti-*D. melanogaster* Vasa antibody was detected using DyLight™647 conjugated affiniPure F(ab')2 fragment goat anti-rat IgM, μ chain specific (Jackson Immunoresearch Labs) and the *S. purpuratus* Vasa antibody was detected using a goat-anti rabbit IgG Alexa Fluor® 488 conjugate (ThermoFisher Scientific). The scale bar is 100 μm.
