# Supplementary material for: Maintenance of somatic tissue regeneration with age in short‐ and long‐lived species of sea urchins
Source: Aging Cell. 2016 Apr 20;15(4):778–87. doi: 10.1111/acel.12487 (PMC4933669; doi:10.1111/acel.12487)
Supplement: Supplementary file 10 — Table S1 Average threshold cycle (Ct) values from qRT‐PCR for cell proliferation, multipotency and control genes in sea urchin tissues. [file ACEL-15-778-s010.pdf]

Table S1 Average threshold cycle (Ct) values from qRT-PCR for cell proliferation, multipotency and control genes in sea urchin tissues (ALM- Aristotle's lantern muscle, ES- esophagus, RN - radial nerve, Coel - coelomocytes, TF - tube feet and spines. Values are Ct ± SEM (n=10 for all tissues except spines which are n=3 for Sp and n=6 for Lv).

A – *S. purpuratus*

	ALM	ES	RN	Coel	TF	Spines
pcna	23.5 ± 0.2	23.1 ± 0.2	24.0 ± 0.2	23.8 ± 0.4	23.7 ± 0.4	23.0 ± 0.1
tert	26.4 ± 0.2	27.2 ± 0.2	27.9 ± 0.2	28.2 ± 0.2	28.9 ± 0.5	28.9 ± 0.3
vasa	24.9 ± 0.4	25.6 ± 0.1	26.7 ± 0.4	24.9 ± 0.3	25.8 ± 0.4	26.1 ± 0.1
seawi	24.7 ± 0.3	25.1 ± 0.1	26.2 ± 0.4	25.2 ± 0.3	26.5 ± 0.7	26.5 ± 0.1
actin	10.6 ± 0.3	16.9 ± 0.1	17.4 ± 0.8	22.4 ± 0.3	16.5 ± 0.5	ND
profilin	18.2 ± 0.3	19.2 ± 0.2	20.2 ± 0.3	19.1 ± 0.3	19.0 ± 0.5	18.1 ± 0.1
cyclophilin	17.4 ± 0.4	17.1 ± 0.2	18.8 ± 0.3	18.8 ± 0.4	18.4 ± 0.4	18.1 ± 0.5
RPL8	18.8 ± 0.2	17.2 ± 0.2	18.8 ± 0.2	17.4 ± 0.4	18.6 ± 0.4	19.0 ± 0.3
ubiquitin	17.6 ± 0.3	18.5 ± 0.3	18.5 ± 0.5	18.4 ± 0.4	21.0 ± 0.5	19.3 ± 0.5

B – *L. variegatus*

	ALM	ES	RN	Coel	TF	Spines
pcna	24.9 ± 0.2	26.2 ± 0.4	26.0 ± 0.7	26.4 ± 0.6	25.8 ± 0.2	26.3 ± 0.4
tert	25.9 ± 0.3	27.6 ± 0.2	29.0 ± 0.4	28.9 ± 0.4	30.1 ± 0.2	29.5 ± 0.4
vasa	26.1 ± 0.2	27.2 ± 0.2	29.6 ± 0.5	29.2 ± 0.5	25.4 ± 0.1	25.2 ± 0.1
seawi	25.6 ± 0.2	26.9 ± 0.2	28.6 ± 0.4	28.1 ± 0.5	28.3 ± 0.4	24.5 ± 0.2
actin	ND	ND	ND	ND	18.7 ± 0.2	31.9 ± 0.6
profilin	20.4 ± 0.1	22.4 ± 0.1	23.8 ± 0.6	24.1 ± 0.6	24.9 ± 0.1	22.5 ± 0.2
cyclophilin	19.6 ± 0.2	18.0 ± 0.2	21.2 ± 0.6	20.6 ± 0.6	23.2 ± 0.4	19.7 ± 0.1
RPL8	18.2 ± 0.2	19.0 ± 0.3	21.8 ± 0.9	23.2 ± 0.8	20.5 ± 0.2	19.7 ± 0.1
ubiquitin	13.7 ± 0.1	15.0 ± 0.1	19.5 ± 0.5	19.0 ± 0.5	20.0 ± 0.4	17.4 ± 0.1
gapdh	22.4 ± 0.4	23.8 ± 0.6		27.0 ± 1.1		

C – *M. franciscanus*

	ALM	ES	RN
pcna	25.4 ± 0.2	25.2 ± 0.4	24.6 ±0.5
tert	27.0 ± 0.2	27.6 ± 0.1	27.7 ± 0.3
vasa	25.0 ± 0.2	26.3 ± 0.1	26.1 ± 0.4
seawi	24.4 ± 0.1	26.0 ± 0.2	26.0 ± 0.2
actin	12.2 ± 0.3	17.0 ± 0.2	16.2 ± 1.2
profilin	25.9 ± 0.2	26.8 ± 0.2	26.9 ± 0.4
cyclophilin	21.5 ± 0.2	22.3 ± 0.2	21.0 ± 0.5
RPL8	20.2 ± 0.1	20.3 ± 0.1	20.8 ± 0.4
ubiquitin	18.7 ± 0.2	19.9 ± 0.2	20.6 ± 0.3
