# Supplementary material for: Maintenance of somatic tissue regeneration with age in short‐ and long‐lived species of sea urchins
Source: Aging Cell. 2016 Apr 20;15(4):778–87. doi: 10.1111/acel.12487 (PMC4933669; doi:10.1111/acel.12487)
Supplement: Supplementary file 11 — Table S2 Expression of nanos2 in tissues of Lytechinus variegatus (Lv), Strongylocentrotus purpuratus (Sp), and Mesocentrotus franciscanus (Mf). [file ACEL-15-778-s011.pdf]

Table S2 Expression of *nanos2* in tissues of *L. variegatus* (Lv), *S. purpuratus* (Sp) and *M. franciscanus* (Mf). Values are given as the threshold cycle (Ct) for qRT-PCR. An average of 2 replicates of pooled cDNA from Lv (n=12), Sp (n=12) and Sf (n=10) were used for Aristotle's lantern muscle (ALM), esophagus (ES), radial nerve (RN) and coelomocytes (Coel). An average of 2 replicates of pooled cDNA from Lv (n=12) and Sp (n=3) tube feet (TF), and Lv (n=8) and Sp (n=3) spines. An average of 2 replicates of pooled cDNA from gonad (male, female and a mixture of male and female, n=2 for all species). Some tissues were not available for testing (ND).

	**Lv – Ct**	**Sp – Ct**	**Mf – Ct**
**ALM**	32.5	32.4	33.6
**ES**	34.5	33.5	32.5
**RN**	30.7	31.5	33.6
**Coel**	32.3	30.7	ND
**TF**	35.3	36	ND
**Spines**	34.9	32.9	ND
**Female Gonad**	20.4	21.2	21.3
**Male Gonad**	24.1	26.0	23.5
**Gonad (mix)**	21.6	22.1	21.5
