# Supplementary material for: Maintenance of somatic tissue regeneration with age in short‐ and long‐lived species of sea urchins
Source: Aging Cell. 2016 Apr 20;15(4):778–87. doi: 10.1111/acel.12487 (PMC4933669; doi:10.1111/acel.12487)
Supplement: Supplementary file 12 — Table S3 qRT‐PCR primer sequences. [file ACEL-15-778-s012.pdf]

Table S3 qRT-PCR primer sequences

Target Gene	GENE ID	Primer Sequences	Product size
Sp, Mf-*pcna*	SPU_023334	(F) GCATTCCAGACACAGAATATGCA<br>(R) GGCTCAGATCTCGACAAATCCT	78
Sp-*tert*	SPU_026612	(F) CTCTGCATCCCCAAAGACAAG<br>(R) GCTAATATGACATGATGGGATTCCT	81
Sp-*seawi*	SPU_023335	(F) CAAGGTCCTATGTAACGAGTCAGTACTAG<br>(R) TCTCGGAACCTGATGTTGTTTG	81
Sp-*vasa*	SPU_008908	(F) GAGGATGCATCAGCACCTTCA<br>(R) CGGGTTGGCGAGACGAT	64
Sp-*nanos2*	DQ286228<br>(GenBank)	(F) AACGGAGAGAGCGAGATGGTAT<br>(R) AGTATCGGGCAGGTTGTGATG	74
Lv-*pcna*		(F) ATGTCATCATTAGCAGCACACTTTAA<br>(R) GACCAATTTAGATGTGATCGCAAT	96
Lv-*tert*		(F) ACGTCGAGTGGACTTCAGAAGAC<br>(R) GCTTGGCCATCTCGATTAACTG	88
Lv-*seawi*		(F) TCCCGAGTTGTGCAGTCGTA<br>(R) GCGTGGCTAGATCCTTCATGA	77
Lv-*vasa*		(F) GGTGAGCGTCAGACCCTGAT<br>(R) CGTTGAGGTACATCCTAGCCTTCT	76
Lv-*nanos*		(F) ACAACAACGTAGTGTCAAATGCAA<br>(R) CGCTCCTCTCATCACTTTCGA	89
Mf-*tert*		(F) GGGTGTGTTCTGTTTGATGTAGCT<br>(R) TCATCAATGTGTACCATCTCCTTCTACT	92
Mf-*seawi*		(F) TGGGTGCTGGCTGCTGTT<br>(R) GCTACAGATGGCTTCCCTTCA	92
Mf-*vasa*		(F) CTCGCAATGTTGGCATCCT<br>(R) CGGACGTGTCGGAAACCT	69
Mf-*nanos*		(F) ACCGGGTTCATGCCATTG<br>(R) CTCATCACCTTGGAGAGCTCAGT	68

Sp Lv Mf *actin1*	SPU_006797	(F) GGTCAGGTCATCACTATTG<br>(R) GCTGTTGTATGTGGTCTC	111
Sp-*ubiquitin*	SPU_021496	(F) ACACTTCATCTTGTTCTCAG<br>(R) GATGGAGTCACTTGGTTC	102
Lv-ubiquitin		(F) CCATCACTCTTGAGGTTGAG<br>(R) AGATGAGACGCTGCTGAT	99
Mf-*ubiquitin*		(F) GGATCTTAGCCTTTACATTCT<br>(R) ACTCTCCATCTCGTTCTC	124
Sp-*profilin*	SPU_020197	(F) GGAAAAAGAAAGGTGAAGGATCAC<br>(R) TGGGCAATGACCGATTACAA	71
Lv-*profilin*		(F) TGCAGGCGAGTAAGACAGCTATA<br>(R) CTCCTTTATTCAAGTTCCCTTGCT	75
Mf-*profilin*		(F) CGCCAACTCCTTTATTTGTATTCC<br>(R) TCTTTTTCCACTCTACAGCGATTG	81
Sp, Mf-*cyc7*	SPU_008305	(F) AGTTTCCATCGTGTCATC<br>(R) CTGTGATCCATTGGTGTT	180
Lv-*cyclophilin7*		(F) CCTCCTTCCACAGGGTTATCC<br>(R) GTACCGTTGCCCCTGGTAAA	67
Sp, Mf-*rpl*8	SPU_010692	(F) GCCATGGTCGGAGTTGTTG<br>(R) TTGCGTTTGACCTTGTACTTGAA	92
Lv-*rpl8*		(F) GCCAACAGGGCCATGGT<br>(R) TTACGCTTGACCTTGTATTTGAAGTAG	104
Lv-*gapdh*		(F) CGAATCACCAAGGAAATC<br>(R) CAGCAGACTCTAAGGAAT	80
